# 4-Thio­carbamoylpyridin-1-ium iodide

**DOI:** 10.1107/S1600536814003511

**Published:** 2014-02-22

**Authors:** Ibukun O. Shotonwa, René T. Boeré

**Affiliations:** a Department of Chemistry and Biochemistry, University of Lethbridge, Lethbridge, AB, T1K 3M4, Canada

## Abstract

The title salt, C_6_H_7_N_2_S^+^·I^−^, crystallizes with two independent cations and two anions in the asymmetric unit. In one of the cations, the dihedral angle between the pyridinium ring and the thioamide group is 28.9 (2)°; in the other it is 33.5 (2)°. In the crystal, N—H⋯S and C—H⋯S hydrogen bonds link the independent cations into pairs. These pairs form a three-dimensional network through additional N—H⋯I and C—H⋯I hydrogen bonds to the anions.

## Related literature   

For details of the synthesis, see: Liebscher & Hartmann (1977 ▶). For related structures, see: Alléaume *et al.* (1973 ▶); Cardoso *et al.* (2008 ▶); Colleter & Gadret (1967 ▶, 1968*a* ▶,*b* ▶); Colleter *et al.* (1970 ▶, 1973 ▶); Gadret & Goursolle (1969 ▶); Gel’mbol’dt, *et al.* (2010 ▶); Kavitha *et al.* (2008 ▶); Revathi *et al.* (2009 ▶). For drug action, see: Vannelli *et al.* (2002 ▶). For a DFT computational study of the parent thio­amide and for vibrational spectroscopy data, see: Wysokińsky *et al.* (2006 ▶).
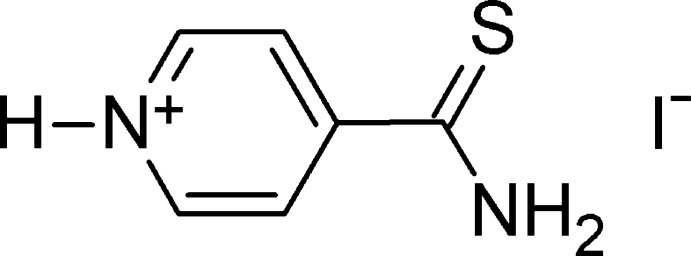


## Experimental   

### 

#### Crystal data  

C_6_H_7_N_2_S^+^·I^−^
*M* *_r_* = 266.10
Monoclinic, 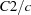

*a* = 18.7580 (11) Å
*b* = 7.7476 (4) Å
*c* = 24.1784 (14) Å
β = 101.165 (1)°
*V* = 3447.3 (3) Å^3^
*Z* = 16
Mo *K*α radiation
μ = 3.89 mm^−1^
*T* = 173 K
0.31 × 0.23 × 0.18 mm

#### Data collection  

Bruker APEXII CCD area-detector diffractometer
Absorption correction: multi-scan (*SADABS*; Bruker, 2008 ▶) *T*_min_ = 0.541, *T*_max_ = 0.746
19180 measured reflections
3923 independent reflections
3684 reflections with *I* > 2σ(*I*)
*R*_int_ = 0.020

#### Refinement  

*R*[*F* ^2^ > 2σ(*F* ^2^)] = 0.014
*wR*(*F* ^2^) = 0.034
*S* = 1.08
3923 reflections
199 parameters
H atoms treated by a mixture of independent and constrained refinement
Δρ_max_ = 0.30 e Å^−3^
Δρ_min_ = −0.36 e Å^−3^

### 

Data collection: *APEX2* (Bruker, 2008 ▶); cell refinement: *SAINT-Plus* (Bruker, 2008 ▶); data reduction: *SAINT-Plus*; program(s) used to solve structure: *SHELXS97* (Sheldrick, 2008 ▶); program(s) used to refine structure: *SHELXL2013* (Sheldrick, 2008 ▶); molecular graphics: *Mercury* (Macrae *et al.*, 2006 ▶); software used to prepare material for publication: *publCIF* (Westrip, 2010 ▶).

## Supplementary Material

Crystal structure: contains datablock(s) I. DOI: 10.1107/S1600536814003511/rn2122sup1.cif

Structure factors: contains datablock(s) I. DOI: 10.1107/S1600536814003511/rn2122Isup2.hkl

Supporting information file. DOI: 10.1107/S1600536814003511/rn2122Isup3.cml

CCDC reference: 987377

Additional supporting information:  crystallographic information; 3D view; checkCIF report

## Figures and Tables

**Table 1:** Hydrogen-bond geometry (Å, °)

*D*—H⋯*A*	*D*—H	H⋯*A*	*D*⋯*A*	*D*—H⋯*A*
N1—H1*A*⋯I2^i^	0.83 (2)	2.79 (2)	3.5996 (18)	164 (2)
N1—H1*B*⋯I1^ii^	0.86 (2)	2.78 (3)	3.6232 (17)	167 (2)
N2—H2⋯I2	0.84 (2)	3.06 (2)	3.6622 (16)	131.0 (18)
N2—H2⋯S2	0.84 (2)	2.71 (2)	3.3410 (16)	133.2 (18)
N3—H3*A*⋯I1^iii^	0.81 (2)	2.86 (2)	3.6188 (17)	157 (2)
N3—H3*B*⋯I2	0.86 (2)	2.73 (3)	3.5854 (18)	173 (2)
N4—H4*A*⋯I1	0.86 (2)	2.69 (2)	3.4804 (17)	152.9 (19)
C4—H4⋯I2^iv^	0.95	3.03	3.8684 (19)	149
C4—H4⋯S2	0.95	2.87	3.4275 (19)	119

Symmetry codes: (i) 
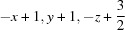
; (ii) 
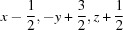
; (iii) 
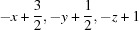
; (iv) 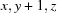.

**Fig. 1.**
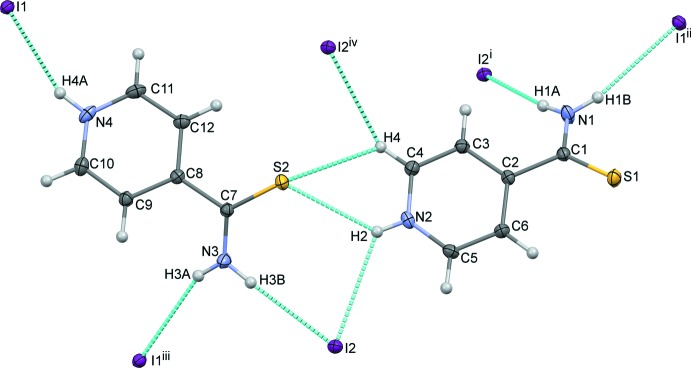
Displacement ellipsoids plot (50% probability) of (I) showing the two independent cations and anions in the asymmetric unit and including additional iodide ions linked by hydrogen bonds; the latter are shown as dashed tubes. [Symmetry codes: (i) -x + 1, y + 1, -z + 3/2; (ii) x - 1/2, -y + 3/2, z + 1/2; (iii) -x + 3/2, -y + 1/2, -z + 1; (iv) x, y + 1, z.]

**Fig. 2.**
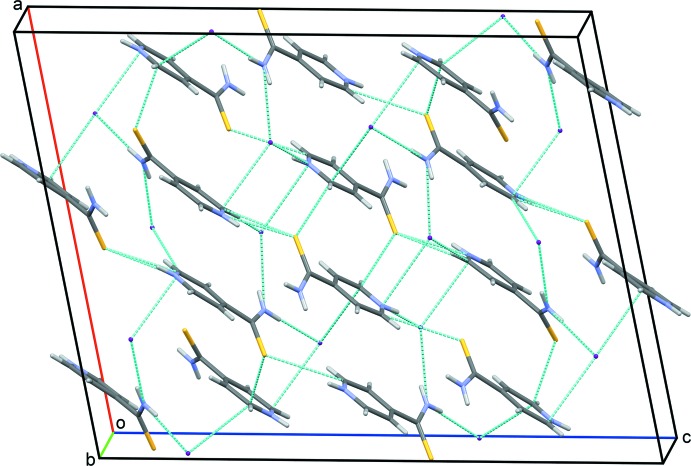
A unit-cell packing diagram viewed down the b axis. The H-bond network is indicated by dashed tubes from H-bond donors to H-bond acceptors.

## supplementary crystallographic information

### 1. Comment

Crystal structures of pyridine rings carrying a thioamide functional group (specifically, 4-thiocarbamoylpyridines) have been investigated primarily because of their use as anti-tuberculosis medications. Thus Colleter and Gadret determined the structure of neutral 4-thiocarbamoylpyridine (Colleter & Gadret, 1967). Ethionamide (2-ethyl-4-thiocaraboylpyridine) is a second line drug used in regimens to treat multi-drug-resistant tuberculosis; its *in vivo* activation has been investigated (Vannelli *et al.*, 2002). The crystal structure of both the HCl and HBr salts of ethionamide have been reported (Colleter & Gadret, 1968*a*, 1968*b*) as has the hexafluorsilicate (Gel'mbol'dt *et al.*, 2010); clinically, ethionamide is administered as the hydrochloride. In order to investigate solubility issues related to drug delivery and the search for similar therapeutic agents, the structures of neutral ethionamide (Alléaume *et al.*, 1973) and the closely related 2-methyl derivative have also been determined (Gadret & Goursolle, 1969). Similarly the 2-propyl (Colleter *et al.*, 1970) and 2-butyl analogues (Colleter *et al.*, 1973) have also been determined as neutral compounds. More recently, the structure of a secondary amine derivative of ethionamide has been determined (Cardoso, *et al.*, 2008). The vibrational spectra of the parent compound have been measured and compared to DFT calculated results (Wysokiński *et al.*, 2006). There is also one reported structure wherein a (substituted) neutral 4-pyridylthioamide coordinates to a copper(II) glyoximato complex (Revathi *et al.*, 2009); this same group earlier undertook the structure determination of the free ligand which is also a secondary amine derivative (Kavitha *et al.*, 2008).

The structure of (I) determined from the X-ray diffraction analysis contains two independent cations and anions (Figure 1). Compared to the parent compound, the average S—C, N2—C and C1—C2 bond distances are shortened in (I); all the other bonds are longer than in the neutral species. There is an extensive network of H-bonds linking the cations and anions in the lattice. One of the independent I^-^ ions interacts with one pyridinium NH donor and two thioamide NH_2_ donors (one from each cation). The second I^-^ interacts strongly with two NH_2_ donors and one of the aromatic ring CH, and only very weakly with a pyridinium NH. In addition, one thioamide S atom is H-bonded to both a pyridinium NH and the *ortho* CH. The strongest N—H···I interaction (donor-acceptor 3.48 (2) Å), which corresponds to 0.49 Å less than the sum of the v.d·W. radii of N and H, is between one of the two I^-^ ions and one of the pyridinium N atoms. In close second place at 0.45 Å less than the sum of the v.d·W. radii is the contact between an NH_2_ nitrogen and the other I^-^ ion, which involves the same thioamide ring. This ring also forms an H-bond with the S atom as acceptor towards the pyridinium NH of the second thioamide in the cluster. In all, there are nine H-bonds which exceed 0.1 Å less than the sums of the corresponding v.d·W. radii.

The H-bonding is comparable in strength (shortest contact 0.49 Å < sum v.d·W. radii) to that reported by Colleter & Gadret (1968*a*, 1968*b*) for the HCl and HBr adducts of ethionamide (shortest contacts 0.34; 0.31 Å < sum of v.d.W. radii). However, compared to these isostructural salts, in (I) the pattern is quite unusual (Figure 2) wherein pyridinium nitrogen N4—H4 are H-bonded to I1, but I2 is linked to the N3 amino rather than to a pyridinium group; the H-bond that forms from this second pyridinium unit involves an S···HN link. There is in fact an extensive network of H-bonds present in this lattice, involving the large iodide anions (Figure 1, Table 1) interacting with N—H and C—H donors. A unit cell packing diagram is provided in Figure 2.

### 2. Experimental

Following a method designed for the oxidation of the thioamide (Liebscher & Hartmann, 1977), 4-pyridine thioamide (1.00 g, 10 mmol) and iodine (2.00 g, 10 mmol) in 7.00 ml glacial acetic acid were heated for about 10 minutes to the boil. The brownish colour of iodine in acetic acid changed to dark red on adding 4-pyridine thioamide. Initially, there were undissolved solids in the mixture, but these dissolved during heating. A dark precipitate was formed upon cooling which was collected on a Buchner funnel and rinsed. After drying on the pump to get rid of acetic acid, the solid was recrystallized from acetonitrile, affording 2.17 g (84%) of dark-brown X-ray quality needles, mp. 426.6–426.8 K. ^1^H NMR, (δ,DMSO): 9.07 p.p.m. (d, 1H, 6.3 Hz), 9.00 p.p.m. (d, 1H, 6.3 Hz), 8.67 p.p.m. (d, 1H, 6.3 Hz), 8.36 p.p.m. (s, 1H, 6.3 Hz), 5.28 p.p.m. (br, 3H + HOD). IR (diamond ATR) (ν, cm^-1^): 3037 w, 1633 m, 1568 w, 1473 s, 1441 m, 1328 w, 1301 m, 1273 m, 1244 m, 1207 m, 1178 m, 1153 m, 1037 m, 844 s, 800 m, 710 s, 627 m, 525 s, 501 s.

### 3. Refinement

Crystal data, data collection and structure refinement details are summarized in Table 1. Crystal data, data collection and structure refinement details are summarized in Table 1. H atoms were located on a difference Fourier map, but for purposes of refinement those bonded to C atoms are treated as riding with C—H(aromatic) = 0.95 Å and *U*_iso_(H) = 1.2*U*_eq_C. H atoms bonded to N atoms in the cations were refined independently but with isotropic displacement parameters set to 1.2*U*_eq_ of the attached N atom.

### Crystal data

**Table:**

C_6_H_7_N_2_S^+^·I^−^	*F*(000) = 2016
*M**_r_* = 266.10	*D*_x_ = 2.051 Mg m^−^^3^
Monoclinic, *C*2/*c*	Mo *K*α radiation, λ = 0.71073 Å
*a* = 18.7580 (11) Å	Cell parameters from 9990 reflections
*b* = 7.7476 (4) Å	θ = 2.5–27.4°
*c* = 24.1784 (14) Å	µ = 3.89 mm^−^^1^
β = 101.165 (1)°	*T* = 173 K
*V* = 3447.3 (3) Å^3^	Block, orange
*Z* = 16	0.31 × 0.23 × 0.18 mm

### Data collection

**Table:**

Bruker APEXII CCD area-detector diffractometer	3923 independent reflections
Radiation source: fine-focus sealed tube, Bruker D8	3684 reflections with *I* > 2σ(*I*)
Graphite monochromator	*R*_int_ = 0.020
Detector resolution: 66.06 pixels mm^-1^	θ_max_ = 27.4°, θ_min_ = 1.7°
φ and ω scans	*h* = −24→24
Absorption correction: multi-scan (*SADABS*; Bruker, 2008)	*k* = −10→10
*T*_min_ = 0.541, *T*_max_ = 0.746	*l* = −31→31
19180 measured reflections	

### Refinement

**Table:**

Refinement on *F*^2^	Primary atom site location: structure-invariant direct methods
Least-squares matrix: full	Secondary atom site location: difference Fourier map
*R*[*F*^2^ > 2σ(*F*^2^)] = 0.014	Hydrogen site location: difference Fourier map
*wR*(*F*^2^) = 0.034	H atoms treated by a mixture of independent and constrained refinement
*S* = 1.08	*w* = 1/[σ^2^(*F*_o_^2^) + (0.014*P*)^2^ + 2.9155*P*] where *P* = (*F*_o_^2^ + 2*F*_c_^2^)/3
3923 reflections	(Δ/σ)_max_ = 0.003
199 parameters	Δρ_max_ = 0.30 e Å^−^^3^
0 restraints	Δρ_min_ = −0.36 e Å^−^^3^

### Special details

**Table:**

Experimental. A crystal coated in Paratone (TM) oil was mounted on the end of a thin glass capillary and cooled in the gas stream of the diffractometer Kryoflex device.
Geometry. All e.s.d.'s (except the e.s.d. in the dihedral angle between two l.s. planes) are estimated using the full covariance matrix. The cell e.s.d.'s are taken into account individually in the estimation of e.s.d.'s in distances, angles and torsion angles; correlations between e.s.d.'s in cell parameters are only used when they are defined by crystal symmetry. An approximate (isotropic) treatment of cell e.s.d.'s is used for estimating e.s.d.'s involving l.s. planes.
Refinement. Amine and pyridinium NH atoms freely refined in order to get H-bond s.u. data. The isotropic displacements were set to 1.2* values of attached N.

### Fractional atomic coordinates and isotropic or equivalent isotropic displacement parameters (Å^2^)

**Table:**

	*x*	*y*	*z*	*U*_iso_*/*U*_eq_	
I1	0.73295 (2)	0.78676 (2)	0.40917 (2)	0.02306 (4)	
I2	0.50416 (2)	−0.34932 (2)	0.65408 (2)	0.02433 (4)	
S1	0.23724 (3)	0.15493 (7)	0.82498 (2)	0.03064 (11)	
N1	0.32690 (10)	0.4190 (2)	0.83135 (8)	0.0322 (4)	
H1A	0.3653 (13)	0.467 (3)	0.8274 (10)	0.039*	
H1B	0.3022 (13)	0.473 (3)	0.8524 (10)	0.039*	
N2	0.42894 (8)	0.0542 (2)	0.69703 (6)	0.0237 (3)	
H2	0.4497 (11)	0.014 (3)	0.6721 (9)	0.028*	
C1	0.30728 (10)	0.2657 (2)	0.81067 (7)	0.0221 (4)	
C2	0.35203 (9)	0.1918 (2)	0.77127 (7)	0.0201 (3)	
C3	0.38691 (10)	0.2986 (2)	0.73855 (8)	0.0226 (4)	
H3	0.3849	0.4204	0.7424	0.027*	
C4	0.42429 (10)	0.2264 (2)	0.70060 (8)	0.0238 (4)	
H4	0.4467	0.2983	0.6771	0.029*	
C5	0.39722 (10)	−0.0526 (2)	0.72831 (8)	0.0258 (4)	
H5	0.4018	−0.1740	0.7247	0.031*	
C6	0.35787 (10)	0.0139 (2)	0.76581 (8)	0.0253 (4)	
H6	0.3348	−0.0613	0.7879	0.030*	
S2	0.48535 (3)	0.15223 (6)	0.57798 (2)	0.02810 (10)	
N3	0.59993 (10)	−0.0467 (2)	0.58319 (8)	0.0304 (4)	
H3A	0.6393 (13)	−0.078 (3)	0.5779 (10)	0.036*	
H3B	0.5804 (13)	−0.120 (3)	0.6025 (10)	0.036*	
N4	0.66095 (9)	0.4143 (2)	0.45727 (7)	0.0294 (4)	
H4A	0.6785 (12)	0.483 (3)	0.4351 (9)	0.035*	
C7	0.56575 (10)	0.0956 (2)	0.56424 (7)	0.0209 (4)	
C8	0.60102 (9)	0.2080 (2)	0.52709 (7)	0.0203 (4)	
C9	0.64262 (10)	0.1370 (2)	0.49061 (8)	0.0244 (4)	
H9	0.6511	0.0161	0.4904	0.029*	
C10	0.67111 (11)	0.2439 (3)	0.45512 (8)	0.0287 (4)	
H10	0.6979	0.1969	0.4291	0.034*	
C11	0.62265 (11)	0.4872 (3)	0.49209 (8)	0.0299 (4)	
H11	0.6173	0.6091	0.4926	0.036*	
C12	0.59099 (10)	0.3860 (2)	0.52727 (8)	0.0259 (4)	
H12	0.5626	0.4369	0.5514	0.031*	

### Atomic displacement parameters (Å^2^)

**Table:**

	*U*^11^	*U*^22^	*U*^33^	*U*^12^	*U*^13^	*U*^23^
I1	0.02298 (6)	0.02070 (6)	0.02617 (6)	−0.00052 (4)	0.00639 (5)	0.00397 (4)
I2	0.02670 (7)	0.01916 (6)	0.02859 (7)	0.00127 (5)	0.00896 (5)	0.00029 (5)
S1	0.0295 (3)	0.0312 (3)	0.0355 (3)	−0.0050 (2)	0.0170 (2)	−0.0023 (2)
N1	0.0314 (9)	0.0302 (10)	0.0400 (10)	−0.0058 (8)	0.0195 (8)	−0.0138 (8)
N2	0.0205 (8)	0.0296 (9)	0.0216 (8)	0.0028 (6)	0.0058 (6)	−0.0056 (6)
C1	0.0227 (9)	0.0233 (9)	0.0211 (8)	0.0025 (7)	0.0060 (7)	0.0004 (7)
C2	0.0185 (8)	0.0228 (9)	0.0186 (8)	0.0012 (7)	0.0029 (7)	−0.0017 (7)
C3	0.0246 (9)	0.0197 (9)	0.0235 (9)	−0.0001 (7)	0.0048 (7)	−0.0003 (7)
C4	0.0246 (9)	0.0248 (9)	0.0226 (9)	−0.0017 (7)	0.0056 (7)	0.0002 (7)
C5	0.0270 (10)	0.0198 (9)	0.0304 (10)	0.0022 (7)	0.0052 (8)	−0.0026 (7)
C6	0.0280 (10)	0.0206 (9)	0.0287 (9)	−0.0006 (8)	0.0086 (8)	0.0014 (7)
S2	0.0268 (2)	0.0295 (3)	0.0317 (2)	0.00596 (19)	0.0148 (2)	0.0049 (2)
N3	0.0259 (9)	0.0295 (9)	0.0392 (10)	0.0061 (7)	0.0150 (8)	0.0130 (8)
N4	0.0259 (8)	0.0325 (9)	0.0284 (8)	−0.0097 (7)	0.0017 (7)	0.0105 (7)
C7	0.0219 (9)	0.0219 (9)	0.0194 (8)	−0.0004 (7)	0.0053 (7)	−0.0006 (7)
C8	0.0185 (8)	0.0213 (9)	0.0204 (8)	−0.0016 (7)	0.0018 (7)	0.0005 (7)
C9	0.0229 (9)	0.0250 (10)	0.0263 (9)	−0.0019 (7)	0.0073 (7)	−0.0004 (7)
C10	0.0260 (10)	0.0353 (11)	0.0261 (9)	−0.0044 (8)	0.0082 (8)	0.0019 (8)
C11	0.0295 (10)	0.0212 (9)	0.0358 (11)	−0.0043 (8)	−0.0020 (8)	0.0061 (8)
C12	0.0278 (10)	0.0225 (9)	0.0267 (9)	−0.0002 (8)	0.0036 (8)	−0.0004 (7)

### Geometric parameters (Å, º)

**Table:**

S1—C1	1.6608 (19)	S2—C7	1.6648 (18)
N1—C1	1.313 (3)	N3—C7	1.312 (2)
N1—H1A	0.83 (2)	N3—H3A	0.81 (2)
N1—H1B	0.86 (2)	N3—H3B	0.86 (2)
N2—C5	1.336 (2)	N4—C11	1.334 (3)
N2—C4	1.341 (2)	N4—C10	1.336 (3)
N2—H2	0.84 (2)	N4—H4A	0.86 (2)
C1—C2	1.500 (2)	C7—C8	1.494 (2)
C2—C6	1.391 (2)	C8—C12	1.392 (3)
C2—C3	1.392 (2)	C8—C9	1.399 (2)
C3—C4	1.377 (3)	C9—C10	1.373 (3)
C3—H3	0.9500	C9—H9	0.9500
C4—H4	0.9500	C10—H10	0.9500
C5—C6	1.376 (3)	C11—C12	1.374 (3)
C5—H5	0.9500	C11—H11	0.9500
C6—H6	0.9500	C12—H12	0.9500
			
C1—N1—H1A	123.0 (17)	C7—N3—H3A	126.7 (17)
C1—N1—H1B	121.3 (16)	C7—N3—H3B	120.9 (16)
H1A—N1—H1B	115 (2)	H3A—N3—H3B	112 (2)
C5—N2—C4	122.57 (16)	C11—N4—C10	122.80 (17)
C5—N2—H2	120.0 (15)	C11—N4—H4A	116.7 (15)
C4—N2—H2	117.2 (15)	C10—N4—H4A	120.5 (15)
N1—C1—C2	115.93 (16)	N3—C7—C8	117.14 (16)
N1—C1—S1	124.24 (14)	N3—C7—S2	123.51 (15)
C2—C1—S1	119.83 (14)	C8—C7—S2	119.32 (13)
C6—C2—C3	118.73 (16)	C12—C8—C9	118.95 (17)
C6—C2—C1	120.18 (16)	C12—C8—C7	119.98 (16)
C3—C2—C1	121.07 (16)	C9—C8—C7	121.04 (16)
C4—C3—C2	119.58 (17)	C10—C9—C8	119.26 (18)
C4—C3—H3	120.2	C10—C9—H9	120.4
C2—C3—H3	120.2	C8—C9—H9	120.4
N2—C4—C3	119.64 (17)	N4—C10—C9	119.73 (18)
N2—C4—H4	120.2	N4—C10—H10	120.1
C3—C4—H4	120.2	C9—C10—H10	120.1
N2—C5—C6	119.70 (17)	N4—C11—C12	119.95 (18)
N2—C5—H5	120.1	N4—C11—H11	120.0
C6—C5—H5	120.1	C12—C11—H11	120.0
C5—C6—C2	119.74 (17)	C11—C12—C8	119.26 (18)
C5—C6—H6	120.1	C11—C12—H12	120.4
C2—C6—H6	120.1	C8—C12—H12	120.4
			
N1—C1—C2—C6	152.73 (19)	N3—C7—C8—C12	−148.94 (18)
S1—C1—C2—C6	−28.0 (2)	S2—C7—C8—C12	32.8 (2)
N1—C1—C2—C3	−28.9 (3)	N3—C7—C8—C9	32.9 (3)
S1—C1—C2—C3	150.45 (15)	S2—C7—C8—C9	−145.37 (15)
C6—C2—C3—C4	1.9 (3)	C12—C8—C9—C10	−1.4 (3)
C1—C2—C3—C4	−176.50 (17)	C7—C8—C9—C10	176.76 (17)
C5—N2—C4—C3	1.4 (3)	C11—N4—C10—C9	−1.3 (3)
C2—C3—C4—N2	−2.4 (3)	C8—C9—C10—N4	2.3 (3)
C4—N2—C5—C6	0.2 (3)	C10—N4—C11—C12	−0.8 (3)
N2—C5—C6—C2	−0.6 (3)	N4—C11—C12—C8	1.7 (3)
C3—C2—C6—C5	−0.4 (3)	C9—C8—C12—C11	−0.6 (3)
C1—C2—C6—C5	178.03 (17)	C7—C8—C12—C11	−178.77 (17)

### Hydrogen-bond geometry (Å, º)

H-bonds link iodide I1 threefold to pyridinium N4 and thioamide NH2 groups N1 and N3. Similarly, I2 is linked to pyridinium N2 and thioamide NH2 groups N1 and N3 (the latter forming an I–N–I–N ring in the lattice) but also involves a (possibly involuntary) short contact to aromatic C4-H. Finally, there are two short contacts lining thioamide S2 with pyridinium N2-H and aromatic C4-H (the latter may also be involuntary.)

**Table:**

*D*—H···*A*	*D*—H	H···*A*	*D*···*A*	*D*—H···*A*
N1—H1*A*···I2^i^	0.83 (2)	2.79 (2)	3.5996 (18)	164 (2)
N1—H1*B*···I1^ii^	0.86 (2)	2.78 (3)	3.6232 (17)	167 (2)
N2—H2···I2	0.84 (2)	3.06 (2)	3.6622 (16)	131.0 (18)
N2—H2···S2	0.84 (2)	2.71 (2)	3.3410 (16)	133.2 (18)
N3—H3*A*···I1^iii^	0.81 (2)	2.86 (2)	3.6188 (17)	157 (2)
N3—H3*B*···I2	0.86 (2)	2.73 (3)	3.5854 (18)	173 (2)
N4—H4*A*···I1	0.86 (2)	2.69 (2)	3.4804 (17)	152.9 (19)
C4—H4···I2^iv^	0.95	3.03	3.8684 (19)	149
C4—H4···S2	0.95	2.87	3.4275 (19)	119

Symmetry codes: (i) −*x*+1, *y*+1, −*z*+3/2; (ii) *x*−1/2, −*y*+3/2, *z*+1/2; (iii) −*x*+3/2, −*y*+1/2, −*z*+1; (iv) *x*, *y*+1, *z*.
